# Application of the sociology theory ethnomethodology to medical education: Utilization of small group learning to combat unconscious bias in patient care

**DOI:** 10.1016/j.amsu.2020.07.007

**Published:** 2020-07-10

**Authors:** Arash Ghaffari-Rafi, Shadeh Ghaffari-Rafi, Rachel Elizabeth Lee, Innocent Aforlabi-Logoh, Andrew Wai Kei Ko, Yohane Gadama, Rana Mehdizadeh, Jose Leon-Rojas

**Affiliations:** a University of Hawaii at Manoa, John A. Burns School of Medicine, Honolulu, HI, USA; b University of Iowa, Carver College of Medicine, Iowa City, IA, USA; c Tamale Teaching Hospital, Tamale, Northern Region, Ghana; d Malawi Liverpool Wellcome Trust Clinical Research Programme, College of Medicine, University of Malawi, Blantyre, Malawi; e University of Queensland, Faculty of Medicine, Brisbane, Australia; f Universidad Internacional del Ecuador Escuela de Medicina, Quito, Ecuador

**Keywords:** Ethnomethodology, Implicit bias, Medical education, PBL (Problem-based learning), Sociology

## Utilization of small group learning to combat unconscious bias in patient care

Notwithstanding a healthcare provider's best intentions, unconscious bias may inadvertently permeate medical practice and impact patient care [1]. Even when assessing a common entity such as pain, racial bias was found not only to be associated with how the clinician perceived a patient's pain, but also accounted for Hispanics and African Americans receiving significantly less pain medication for equivalent health issues than White patients [1,2]. Overall, numerous health disparities have been elucidated to have roots in providers unconsciously stereotyping, despite best interest in patient wellbeing [[1], [2], [3]].

Although medical institutions have sought to counter implicit bias by increasing class diversity, even amongst a diverse class, students of similar backgrounds tend to gravitate towards and form closest bonds with those possessing similar identities (political, socioeconomic, race, religion, sexual orientation, etc.), and thus interact less with differing perspectives [[4], [5], [6], [7]]. In turn, students have less exposure to differing viewpoints, and thus decreased ability to become cognizant of one's biases towards the “other” [[4], [5], [6]]. One potential route for reducing biases in patient care, is application of sociologic theory to the medical curriculum, specifically ethnomethodology [8,9].

Ethnomethodology explains how one sustains a reality—reality being a social construct permitting humans to make meaning of the environment (i.e., interactions, events) [8,10]. The theory contends there are five features to every reality: *reflexivity*, *coherence*, *interaction*, *fragility*, and *permeability* [[8], [9], [10]]. Unconscious bias being a specific reality (worldview) of the beholder, will thus exhibit all five features of reality [10].

*Reflexivity*, the first feature, asserts that once one holds a belief about the world, all experiences (including counterevidence) will serve to support the belief [10,11]. The belief is termed “an incorrigible proposition,” defined as a firm fundamental perspective one maintains about the world, which can never be proven wrong to the believer, as counterevidence will be ignored or rationalized [8,10,11]. In subsets of the healthcare provider population, existence of incorrigible propositions contributes to sustenance of stigmas towards various diseases (tuberculosis, leprosy, sexually transmitted diseases) and procedures (abortion or cosmetic surgery) [[12], [13], [14], [15], [16], [17]]. Likewise, incorrigible propositions partially account for why coronary artery disease is treated less aggressively amongst women and minority ethnicities [1,3,18].

*Coherence*, the second feature of reality, contends all parts of the belief system or body of knowledge, will support each other without exception [8,10]. The believer's reality is a group of ideas which logically fit together, systematically making sense of the world [[8], [9], [10]]. When facing contradictions, the believer uses a rationalization to support the held biases [[8], [9], [10]].

Furthermore, reality is constructed through *interaction*—the one feature which can be influenced by a medical curriculum [8,10,11]. The social network of the bias-possessor will provide the interactions to create one's reality, for humans develop meaning through communication [4,5,8,10]. Hence, if a medical student is submerged in a setting requiring interaction with five to eight classmates of different backgrounds, those interactions will help shape an alternative reality for the student, which will then bring unconscious biases to awareness [8,10].

As every reality is *fragile*, there exists a possibility for beliefs to alter if assumptions that establish reality are undermined [8,10]. Therefore, any unconscious bias of the medical student can be modified if undermined by proper social interactions with classmates possessing differing perspectives [10].

*Permeability*, the fifth feature, indicates reality can alter, upon meeting three conditions: the student has no place to escape, no time to escape, and no one to provide counterevidence—an isolationist environment [6]. Permeability expects the subject to step outside the comfort zone [10]. By placing medical students in diverse work groups where classmates hold opposing perspectives (religious, cultural, political, etc.), students will be placed outside their comfort zones, and for that class period, have no place and time to escape, with potentially no one to provide counterevidence. In ethnomethodology, creating an isolationist environment highlights a feasible solution for developing self-awareness of biases and new perspectives [8,10,11]. However, establishing the three conditions for permeability is more feasible in a problem-based learning (PBL) curriculum than lecture-centric [19].

Advanced and led by the University of Hawaii in the 1980s, PBL involves learning within small diverse groups of five to eight students with differing backgrounds randomly placed together for a curricular unit [19]. Therefore, PBL learning can tacitly coerce students to deeply communicate with colleagues one typically would not intensely interact with [19]. By creating a class dynamic where students are placed in situations necessitating communication and collaboration with everyone in the immediate surrounding, such will potentially provide memorable and personal encounters with classmates considered as the “other; ” hence actively teaching students to be aware of their unconscious biases.

Overall, in training the next generation of healthcare practitioners, medical schools should recognize not only are clinicians required to utilize subjective perception in practice, but also that most humans perceive each other's difference within milliseconds of visualization, consequently to address health disparities promulgated via unconscious biases there should be a substantial effort to engage such issues [20]. Without confronting the realities of unconscious biases, patients' lives will continue to be impacted. Although some outcomes will be innocuous, others more deleterious—including minorities continuing to be less likely provided the proper cardiac medications, undergo cardiac bypass surgery, begin kidney dialysis or attain kidney transplants, while more often attaining life-restricting procedures such as lower-limb amputations for diabetes and other diseases [[1], [2], [3]]. Therefore, application of ethnomethodology to medical curriculum in small-group learning, provides one avenue for better engaging implicit bias, and thus improving patient outcomes.

## Provenance and peer review

Not commissioned, externally peer reviewed.

## Ethical approval

No applicable. All ethnical standards were meet for this manuscript (perspective piece).

## Please state any sources of funding for your research

No sources of funding.

## Author contribution

All authors contributed to development of this manuscript.

## Registration of research studies

1. Name of the registry: Not applicable
2. Unique Identifying number or registration ID: Not applicable
3. Hyperlink to your specific registration (must be publicly accessible and will be checked): Not applicable.

## Guarantor

No data was involved with this study. But the Guarantors are Arash Ghaffari-Rafi, Shadeh Ghaffari-Rafi, Rachel Elizabeth Lee, Innocent Aforlabi-Logoh, Andrew Wai Kei Ko, Yohane Gadama, Rana Mehdizadeh, Jose Leon-Rojas.

## Consent

No patients, volunteers, or animals were utilized in this study; not applicable.

## Disclosures

None.

## Declaration of competing interest

All authors declare **no conflicts of interest.**
